# Distribution of pelagic squids *Abraliopsis* Joubin, 1896 (Enoploteuthidae) and *Pterygioteuthis* P. Fischer, 1896 (Pyroteuthidae) (Cephalopoda, Decapodiformes, Oegopsida) in the Mexican Pacific

**DOI:** 10.3897/zookeys.537.6023

**Published:** 2015-11-18

**Authors:** Michel E. Hendrickx, Brian Urbano, Pablo Zamorano

**Affiliations:** 1 Laboratorio de Invertebrados Bentónicos, Unidad Académica Mazatlán, Instituto de Ciencias del Mar y Limnología, Universidad Nacional Autónoma de México PO, Box 811, Mazatlán, Sinaloa, 82000, Mexico; 2 Facultad de Ciencias, Universidad Nacional Autónoma de México, Ciudad Universitaria, Av. Universidad, 3000, 04510, México D.F., Mexico; 3 Delegación Federal de la SEMARNAT en el Estado de Colima Victoria 360, Col. Centro, Colima, Col. C.P. 28000

**Keywords:** Mexican Pacific, squids, *Abraliopsis*, *Pterygioteuthis*, distribution, TALUD cruises

## Abstract

The oegopsid squids *Abraliopsis* and *Pterygioteuthis* are abundant and diverse genera with taxonomic and distributional problems. Identification and distribution of species in the Mexican Pacific has been somewhat controversial. Here are provided a large series of new records for *Abraliopsis affinis*, *Abraliopsis falco*, *Pterygioteuthis gemmata*, *Pterygioteuthis giardi* and *Pterygioteuthis hoylei* from the Gulf of California and off the SW coast of Mexico. All five species were collected in the central or the southern Gulf of California, or in both. *Abraliopsis affinis* was found in seven samples with a total of 48 specimens, from 21°59' to 24°53'12"N. *Abraliopsis falco* was much less represented in the samples (14 specimens) but it was found in 10 localities, four of which correspond to the central-southern Gulf of California (north to 27°44'53"N) and six to SW Mexico (south to 16°49'18"N). In the case of *Pterygioteuthis gemmata*, only two records (three specimens) were obtained, both in the SW Gulf of California, while *Pterygioteuthis giardi* (nine specimens) records were all from the central Gulf of California (27°44'53” to 25°39'59"N). In the case of *Pterygioteuthis hoylei* (nine specimens), material was obtained in six localities, also in a restricted latitudinal range (24°23'48” to 25°56'56"N).

## Introduction

Cephalopoda is a major group of marine mollusks with almost 1000 species worldwide (Roper et al. 1995). An important component of the natural communities, they are active predators mostly on invertebrates (e.g., mollusks, crustaceans) and fishes. They are also used as prey for many medium to large size marine species (e.g., fish, marine mammals, sea birds) and therefore occupy an important position in the marine food web (Boyle and Rodhouse 2005). They also represent a significant portion of the worldwide catch of marine products, either by the fishing fleets or by fisherman in coastal, shallow water (Jereb et al. 2014).

Cephalopods are essentially divided into two natural groups: the pelagic forms that permanently swim into the water column (i.e., squids, nautilus or cuttlefishes) and the benthic species, that live on or close to the bottom (i.e., octopuses) (Strugnell et al. 2005). Our knowledge on distribution, ecology and biology of small pelagic squids, their larvae, paralarvae and juvenile phases are very limited, particularly in tropical oceans (Vidal et al. 2010; Alejo-Plata et al. 2013). Pelagic cephalopods are fast-moving animals and are able to detect the approach of sampling gears either by vision or detection of vibrations (Boyle and Boletzky 1996). They are therefore very effective at avoiding nets (Lansdell and Young 2007). The use of large sampling gear like the RTM8 deployed off the Brazilian coast (Vidal et al. 2010) has proved very effective at capturing small cephalopods, thus increasing the potential for their study.

Within the pelagic forms there is a general agreement to recognize two groups: 1) the Myopsida squids, which contains mainly the loliginids species, and 2) the Oegopsida, a more diverse and rich group characterized by the presence of an ocular membrane (Jereb and Roper 2010). At present, the Oegopsida is composed by 24 families and contains very large and very small species, some living in very deep water. It also contains some commercial species. The “Enoploteuthidae” is a group forming a clade of closely-related families, all of which are of small size (< 5 cm), live in the mesopelagic zone, and possess a large amount of photophores along their entire body (Young et al. 2012).

According to Young et al. (1998) 12 species of *Abraliopsis* Joubin, 1896 (type species: *Abraliopsis pfefferi* Joubin, 1896) are known worlwide, while Roper and Jereb (2010) included only 11 species of *Abrialopsis* in their list. According to Bouchet and Gofas (2014) the genus *Abraliopsis* contains 12 species. Bouchet and Gofas (2014), however, indicated that the status of several species is somewhat imprecise. According to Young and Tsuchiya (2014) there are approximately twenty species of *Abraliopsis* worldwide, of which ten are undescribed. These authors presented a tentative list of taxonomic features that allows the separation in four genera of *Abraliopsis braliopsis*, but they indicated that further study is in need before a final decision is taken in dividing the genus. Only three species of *Abraliopsis* have been reported in the eastern Pacific: *Abraliopsis affinis* (Pfeffer, 1912) *Abraliopsis falco* Young, 1972, and *Abraliopsis felis* McGowan & Okutani, 1968 (Young et al. 1998; Roper and Jereb 2010). *Abraliopsis affinis* occurs in the tropical waters of the eastern Pacific Ocean, and is known from Chile, Colombia, Costa Rica, Ecuador, El Salvador, Guatemala, Honduras, Mexico, Nicaragua, Panama and Peru. *Abraliopsis falco* has been reported from off the Baja California Peninsula (type locality and only area where it has been found so far) (Young et al. 1998; Abitia Cardenas et al. 2011). The third species, *Abraliopsis felis*, is found off the west coast of North America, from about 27° to 43°N (Young et al. 1998). Species are separated by the number, size, and position of photophores, and by the number, size and distribution of the hooks and suckers on the tentacles (Sweeney et al. 1992).

The genus *Pterygioteuthis* P. Fischer, 1896 (type species *Pterygioteuthis giardi*, P. Fischer, 1896) contains another group of small pelagic squids with a worldwide distribution. Young et al. (1998) considered three species within the genus *Pterygioteuthis*: *Pterygioteuthis giardi*, *Pterygioteuthis gemmata* Chun, 1908, and *Pterygioteuthis microlampas* Berry, 1913. They further indicated that two subspecies of *Pterygioteuthis giardi* have been recognized: the nominal subspecies, occurring in the Atlantic (Diekman et al. 2002) and the Indo-West Pacific, and *Pterygioteuthis giardi holyei* Pfeffer, 1912 from the tropical eastern Pacific. The eastern Pacific subspecies was elevated to genus based on a detailed morphological study (Lindgren 2010). A detailed study of the distribution of *Pterygioteuthis hoylei* in the Gulf of California (including paralarvae and adults) was provided by De Silva-Dávila et al. (2013). Lindgren (2010) also analyzed the distribution of other species of *Pterygioteuthis* in the west central and eastern Pacific, noting that only *Pterygioteuthis gemmata* (Indo-West Pacific, eastern Pacific and Atlantic) and *Pterygioteuthis hoylei* (eastern Pacific and Equatorial Countercurrent to about 125°W) are present in the eastern Pacific. Members of this genus are most commonly collected in mid water surveys, specially the paralarvae, while adult specimens are usually not very abundant in the samples (Bowerl et al. 1999).

Together with fishes, stomatopods and benthopelagic shrimps, species of *Abraliopsis* (e.g., *Abraliopsis pacifica* Tsuchiya & Okutani, 1990) are an important component of the micronecton near seamounts (Drazen et al. 2011). Juveniles and adults of small squids are also important prey items for many species of pelagic fishes and some marine mammals (Fiscus et al. 1989). Specimens of *Abraliopsis affinis* have been found to be part of the diet of the Peruvian hake (*Merluccius gayi peruanus* Ginsburg, 1954) (Blaskovic 2011), of the striped marlin *Kajikia audax* (Philippi, 1887) from off Cabo San Lucas (Abitia Cardenas et al. 2011), and of sharks in the Ecuadorian and Mexican Pacific (Galván-Magaña et al. 2013). *Abraliopsis* sp. was found in stomach content of the Indo-Pacific sailfish, *Istiophorus platypterus* (Shaw, 1792) (Varghese et al. 2013). Other records include specimens of *Abraliopsis falco* Young, 1972 and *Abraliopsis* spp. found as part of the diet of the yellowfin tuna *Thunnus albacares* (Bonnaterre, 1788) in the eastern tropical Pacific (Olson et al. 2014). *Abraliopsis lineata* (Goodrich, 1896) is part of the diet of the common dolphinfish, *Coryphaena hippurus* Linnaeus, 1758, in the eastern Arabian Sea (Varghese et al. 2013). Enoploteuthidae constituted over 25% of estimated biomass of squids consumed by a specimen of the pygmy sperm whale *Kogia breviceps* (de Blainville, 1838), stranded on a beach in Tasmania (Beasley et al. 2013). Although not fully identified, *Abraliopsis gilchristi* Robson, 1924 and *Enoploteuthis galaxias* Berry, 1918 were probably the two species of Enoploteuthidae found in the stomach content of this whale. Specimens of *Abraliopsis* have also been found as an important item in the diet of the shrimp *Aristaeomorpha foliacea* (Risso, 1827) in the Mediterranean Sea, with *Abraliopsis pfefferi* (Robson, 1924) representing the dominant species of cephalopods in the diet (Markaida and Sosa-Nishizaki 2003). *Pterygioteuthis* species are also consumed by many marine animals, including a large variety of fish (Ménard et al. 2013), other cephalopods like *Dosidicus gigas* (D'Orbigny, 1835) (Camarillo-Coop et al. 2013), sea-birds like the shearwater *Puffinus newelli* Henshaw, 1900 (Ainley et al. 2014), and fur seal (*Arctocephalus townsendi* Merriam, 1897) (Gallo-Reynoso and Esperón-Rodríguez 2013).

There have been several studies on the occurrence of paralarvae and small juveniles of cephalopods. They are known to be related to primary production in upwelling areas (Vidal et al. 2010) and their abundance is related with temperature, especially in areas that experience fast and significant temperature changes (Vecchione 1999). Some of these recent studies deal with the eastern Pacific. Okutani and Mcgowan (1969) and Watson and Manion (2011) reported on paralarvae from the California Current, including species of *Abraliopsis* and *Pterygioteuthis*. Granados-Amores et al. (2010) studied the paralarvae of cephalopods collected off the west coast of the Baja California Peninsula. Their samples included 10 families and 17 species (plus some unidentified species). The Enoploteuthidae included at least three species of unidentified *Abraliopsis* and *Abraliopsis felis*, while the Pyroteuthidae included two unidentified species of *Pterygioteuthis*. Alejo-Plata et al. (2013) recorded cephalopods paralarvae and juveniles in the Gulf of Tehuantepec, noting that six families representing eight genera and at least 13 species were present in the samples. Enoploteuthidae represented 15.9% of specimens and comprise of three unidentified species of *Abraliopsis* and unidentifed material. A very complete study of distribution and abundance of *Pterygioteuthis hoylei* was performed by De Silva-Dávila et al. (2013) in the Gulf of California. Based on 241 plankton samples, they were able to identify most paralarvae using COI barcode information available in the Gen Bank. Their distribution data indicated that *Pterygioteuthis hoylei* occurs from the southern Gulf of California up to ca 20°30'N.

During an intensive survey of the deep-water fauna inhabiting below the Oxygen Minimum Zone (OMZ), the TALUD project, specimens of small squids were collected in different sampling gear off the coast of western Mexico. This material belongs to the genera *Pterygioteuthis* and *Abraliopsis* and is reported herein.

## Material and methods

The material on which this study is based was collected by the R/V “El Puma” of the Universidad Nacional Autónoma de México (UNAM), between 1991 and 2014. Specimens of pelagic squids were captured during sampling operations in the Gulf of California (a total of eight cruises: TALUD III, September 1991; TALUD IV, August 2000; TALUD VII, June 2001; TALUD VIII, April 2005; TALUD IX, November 2005; TALUD X, February 2007; TALUD XIII, January 2009) and off the SW coast of Mexico, from Jalisco to Guerrero (TALUD XII, March-April 2008). During these cruises, a total of 113 localities, from 216 to 2300 m depth, were sampled for benthic species. Positional coordinates for each sampling station were obtained using a GPS navigation system. Depth was measured with an EdoWestern analogic recorder (TALUD III-VIII) or a digital recorder (TALUD IX-XIII). All the specimens were presumably captured during the ascent of a modified Agassiz dredge (2.5 m width, 1 m high) and a standard benthic sledge (2.35 m width, 0.9 m high) equipped with a modified shrimp net (ca. 5.5 cm stretched mesh size) with a ca. 2.0 cm (3/4”) internal lining net. In these cases, the depth range at which the gear was operated is provided but does not indicate the depth of capture because the specimens could have been captured between surface and maximum trawling depth at each locality. In two ocasions, sample were obtained with a micronecton net or a mid water Issacs-Kidd trawl. The material collected during this survey is deposited in the Regional Collection of Marine Invertebrates (EMU), at UNAM in Mazatlán, Mexico. The size (mantle length) was measured to the nearest 0.1 mm. Abbreviations are: St., sampling station; ML, mantel length; AD, Agassiz dredge; BS, benthic sledge. Systematic sequence used herein is according to Young et al (2012).

## Results

Five species of small squids were collected during sampling operations. A total of 86 specimens were obtained: *Abraliopsis affinis*, 46; *Abraliopsis falco*, 18; *Pterygioteuthis gemmata*, 3; *Pterygioteuthis giardi*, 9; and *Pterygioteuthis hoylei*, 10.

### Systematic section

**Cephalopoda**

**Class Coleoidea**

**Superorder Decapodiformes**

**Order Oegopsida**

**Family Enoploteuthidae Pfeffer, 1900**

***Abraliopsis* Joubin, 1896**

***Abraliopsis affinis* (Pfeffer, 1912)**

**Material examined.** TALUD III. St. 3B (22°36'36"N; 106°35'54"W), Aug 17, 1991, 1 org. (ML 26.5 mm), Issac Kidds mid-water trawl, 275 m (total depth, 940–950 m) (EMU-10591); St. 14A (24°38'48"N; 108°26'54"W), Aug 19, 1991, 24 orgs. (ML 22.3–34.7 mm), AD operated at 1016–1020 m (EMU-10592). TALUD IV. St. 4 (21°59'N; 106°35'W), Aug 23, 2000, 15 orgs. (ML 20.1–32.1 mm), AD operated at 1200–1290 m (EMU-10593); St. 13 (23°17'30"N; 107°29'51"W), Aug 24, 2000, 1 org. (ML 18.9 mm), BS operated at 860 m (EMU-10597). TALUD VII. St. 12 (23°18'18"N; 107°26'48"W), Jun 6, 2001, 2 orgs. (ML 25.2–27.8 mm), AD operated at 1040–1120 m (EMU-10595); St. 18 (24°14'30"N; 108°16'24"W), Jun 7, 2001, 2 orgs. (ML 27.8–32.1 mm), AD operated at 1040–1120 m (EMU-10596). TALUD VIII. St 21 (26°2'18"N; 110°37'6"W), Apr 19, 2005, 1 org. (ML 22.4 mm), AD operated at 1380 m (EMU 10598).

***Abraliopsis falco* Young, 1972**

**Material examined.** TALUD IV. St. 25 (24°53'12"N; 108°59'24"W), Aug 26, 2000, 2 orgs. (ML 25.0 mm), AD operated at 778–800 m (EMU-10594). TALUD V, St. 3 (21°59'14"N; 106°28'30"W), Dec 13, 2000, 1 org. (ML 23.5 mm), BS operated at 730 m (EMU-10599). TALUD VII, St. 26 (24°25'24"N; 109°05'21"W), Jun 8, 2001, 1 org. (ML 32.2 mm), BS operated at 1180–1260 m (EMU-10600). TALUD X. St. 14 (27°44'53"N; 111°39'54"W). Feb 11, 2007, 1 org. (ML 24.2 mm), BS operated at 843–905 m (EMU-10601). TALUD XII. St. 2 (16°49'18"N; 100°30'52"W), Mar 28, 2008, 1 org. (ML 21.3 mm), BS operated at 990–1088 m (EMU-10602); St 3 (16°54'35"N; 100°44'10"W), Mar 28, 2008, 2 orgs. (ML 27.1–27.6 mm), BS operated at 1380–1456 m (EMU-10603); St. 10 (17°11'03"N; 101°28'05"W), Mar 28, 2008, 1 org. (ML 32.6 mm), BS operated at 1180–1299 m (EMU-10604); St. 29 (19°19'37"N; 105°26'20"W), Apr 2, 2008, 1 org. (ML 25.4 mm), BS operated at 1609–-1643 m (EMU-10605); St. 30 (19°22'05"N; 105°16'18"W), Mar 28, 2008, 4 orgs. (ML 27.8–-29.3 mm), BS operated at 1350–-1380 m (EMU-10606); St. 30B (19°30'37"N; 105°19'16"W), Mar 26, 2008, 3 orgs. (ML 25.7–30.3 mm), BS operated at 865–1045 m (EMU-10607). TALUD XIII. St. B (26°19'54"N; 110°29'12"W), Jan 13, 2009, 1 org. (ML 30.8 mm), Agassiz dredge, 1295–1330 m (EMU-10608).

**Family Pyroteuthidae Pfeffer, 1912**

***Pterygioteuthis* P. Fischer, 1896**

***Pterygioteuthis gemmata* Chun, 1908**

**Material examined.** TALUD VIII. St 15 (25°21'27"N; 110°18'18"W), April 18, 2005, 2 orgs. (ML 12–16 mm), BS operated at 2100 m (EMU-10609); St. 21 (26°02'18"N, 110°37'06"W), April 19, 2005, 1 org. (ML 13 mm), BS operated at 1380 m (EMU-1598).

***Pterygioteuthis giardi* P. Fischer, 1896**

**Material examined.** TALUD IV. St. 35 (25°39'59"N; 110°11'17"W). Aug 27, 2000, 1 org. (ML 20.6 mm), BS operated at 2000–2100 m (EMU-10615). TALUD VIII. St. 18 (25°50'N; 110°34'W), Feb 12, 2005, 3 orgs. (ML 15.5–17.7 mm), micronecton net, 690 m (total depth 1300 m) (EMU-10616). TALUD IX. St. 22 (26°3'42"N; 110°20'36"W), Nov 14, 2005, 1 org. (ML 19.4 mm), BS operated at 2214–2309 m (EMU-10617). TALUD X. St. 14 (27°44'53"N; 111°36'58"W), 3 orgs. (ML 17.8–19.1 mm), BS operated at 905–943 m (EMU-10618). TALUD XIII, St. 36 (26°07'12"N 110°30'53"W), Jan 15, 2009, 1 org. (ML 18.6 mm), AD operated at 2300–2360 m (EMU-10619).

***Pterygioteuthis hoylei* Pfeffer, 1912**

**Material examined.** TALUD VIII. St. 15 (25°23'06"N; 110°18'24"W), Abr 18, 2005, 2 orgs. (ML 12.1–12.2 mm), BS operated at 2100 m (EMU-10609); St. 20 (25°56'56"N; 110°43'W), Abr 19, 2005, 2 orgs. (ML 16.2–18.0 mm), BS operated at 1140–1150 m (EMU-10610). TALUD IX. St. 8 (25°07'28"N; 109°49'48"W), Nov 12, 2005, 1 org. (ML 17.6 mm), BS operated at 1657 m (EMU-10611); St.15 (25°21'27"N; 110°18'18"W), Nov 13, 2005, 2 orgs. (ML 19.2 mm), BS operated at 1985–2290 m (EMU-10612); St. 16 (24°23'48"N; 110°36'42"W), Nov 13, 2005, 1 org. (ML 16.4 mm), BS operated at 997–1021 m (EMU-10613); St. 20B (25°58'7"N; 110°40'4"W), Nov 14, 2005, 2 orgs. (ML 17.4–19.6 mm), BS operated at 1229–1343 m (EMU-10614).

## Distribution

**Figure 1.**
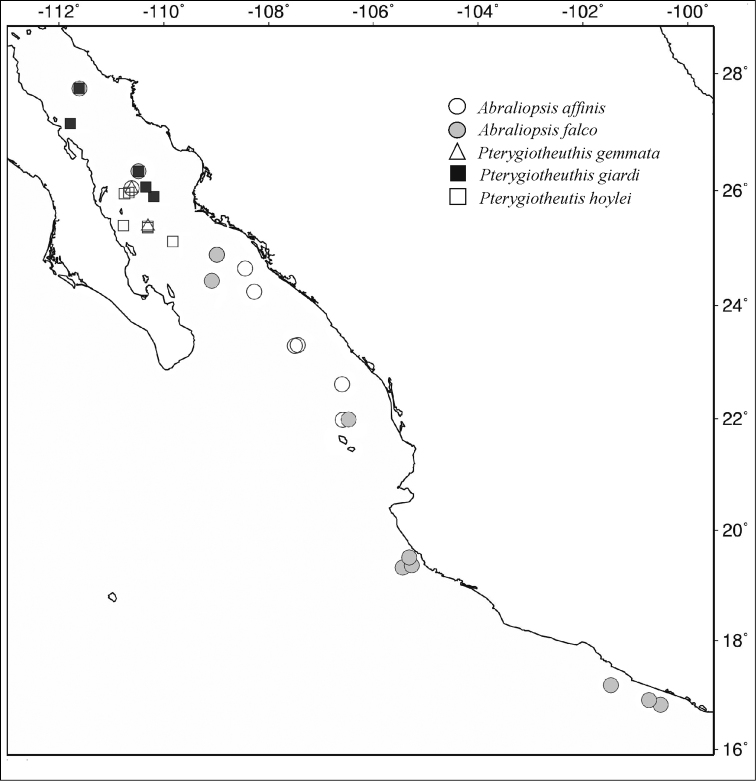
Distribution of specimens of *Abraliopsis affinis* (Pfeffer, 1912), *Abraliopsis falco* Young, 1972, *Pterygioteuthis gemmata* Chun, 1908; *Pterygioteuthis giardi*, P. Fischer, 1896, and *Pterygioteuthis hoylei* Pfeffer, 1912 collected during the TALUD cruises off the Pacific coast of Mexico.

All five species examined herein were collected in the central or the southern Gulf of California, or in both (Fig. 1). *Abraliopsis affinis* was found in seven samples with a total of 48 specimens, from 21°59' to 24°53'12"N. *Abraliopsis falco* was much less represented in the samples (14 specimens) but it was found in 10 localities, four of which correspond to the central-southern Gulf of California (north to 27°44'53"N) and six to SW Mexico (south to 16°49'18"N), thus covering a much wider latitudinal range than the other three species (Fig. 1). *Pterygioteuthis gemmata* (3 specimens) was collected in two stations located in the SW Gulf of California (Fig. 1). In the case of *Pterygioteuthis giardi* (9 specimens), records are only from the central Gulf of California (27°44'53” to 25°39'59"N) and, in the case of *Pterygioteuthis hoylei* (9 specimens), material was obtained in six localities, also in a restricted latitudinal range (24°23'48" to 25°56'56"N) (Fig. 1).

## Discussion

In this study the material was collected either during the ascending process of large gear used for sampling the benthos or with a micronecton net and a mid-water trawl (two samples only). Although the benthic samplers were not designed to catch small pelagic squids, a rather large series of specimens was collected over the study period. In spite of this, the two oegopsid species were very common in the samples obtained during the TALUD survey (*Abraliopsis affinis*, 46; *Abraliopsis falco*, 18). The pyrotheutids were much less abundant in the samples (*Pterygioteuthis gemmata*, 3; *Pterygioteuthis giardi*, 9; *Pterygioteuthis hoylei*, 10). However, it was decided not to evaluate density of species collected in each sample due to the fact that many specimens were probably able to avoid the nets.

Two species of *Abraliopsis* were collected during the TALUD survey: *Abraliopsis affinis* and *Abraliopsis falco*. *Abraliopsis felis* has been reported from the NE Pacific, between 27° and 43°N (Young et al. 1998), but was not found during our survey. *Abraliopsis falco* type material was collected by the “*Velero IV*” from off the Baja California Peninsula, and it has not been reported from outside this area so far (Young et al. 1998). Our records indicate that this species has a wider distribution range than previously thought. More samples are needed, however, to define its exact distribution range. It was not found in the Gulf of Tehuantepec by Alejo-Plata et al. (2014), who only reported *Abraliopsis affinis* from that area. During the present survey *Abraliopsis falco* was collected in the southern Gulf of California (northernmost limit set at 27°44'53"N, 111°39'54"W) and extends its distribution south to 16°49'18"N (Fig. 1). Although it also appears to be endemic to the eastern Pacific, *Abraliopsis affinis* had a much wider distribution range, from Ecuador (02°34'N) to Mexico (14°46'N) (Young et al. 1998). Our records indicate that it extends much further north, entering the Gulf of California where its northernmost distribution limit is set at 26°2'18"N, 110°37'6"W. Young (1972) indicated that the presence of *Abraliopsis falco* is strongly correlated with high salinity water masses, and the Gulf of California water is highly saline (≥ 35 ppm) (Alvarez-Borrego 2010). Species of *Abraliopsis* are reported as diurnal migrants, spending most of the day between 300 and 600 m (Roper and Young 1975). Most of our samples, however, were made in water deeper that 600 m, thus crossing the entire depth interval where these species are supposed to occur and much more (maximum depth reached by the gear, 2300 m).

Lindgren (2010) reviewed the status of the species of *Pterygioteuthis* occurring in the eastern and eastern-central Pacific, noting that *Pterygioteuthis giardi hoylei* (sensu Young et al. 1998) *Pterygioteuthis giardi*, *Pterygioteuthis gemmata*, and *Pterygioteuthis microlampas* occur roughly between the Equator (ca. 5°S) and 32°N. Based on morphological analysis, Lindgren (2010) upgraded *Pterygioteuthis giardi hoylei* to full-species rank. The two species were distinguished based on several characters, including the size and numbers of hooks on arm I, the presence or absence of suckers on male arm III, the extension of chromatophores on tentacle stalk, the presence or absence of rows of chromatophores on aboral tentacle club, and on numbers and position of chromatophores on funnel and tentacles. Lindgren (2010) defined a restricted distribution of *Pterygioteuthis giardi* in the eastern-central Pacific (not closer to the continent than 140°W), but emphasized that further sampling in the area might demonstrate that it extends further to the east, and would therefore share a large distribution range with *Pterygioteuthis hoylei*. In the case of *Pterygioteuthis gemmata*, morphologically indistinguishable populations occur in the Atlantic (type locality in the South Atlantic), the Indo-Pacific and the eastern Pacific (Lindgren 2010).

Our records show that *Pterygioteuthis gemmata*, *Pterygioteuthis hoylei* and *Pterygioteuthis giardi* all occur in the southern Gulf of California. The bathymetric distribution and the southern distribution limit of *Pterygioteuthis gemmata* are unclear. Lindgren (2010) reported the presence of this species from off California, USA, and off Baja California (27°26'N to 32°55'N), in Pacific Mexico. Our records appears to be the first available for the Gulf of California, and would represent the current southernmost distribution limit of the species in the eastern Pacific. *Pterygioteuthis hoylei* is restricted to the Gulf of California, the coastal area of Central America and to an offshore area extending from the Galapagos Islands to ca. 145°W, roughly matching the extension of the Oxygen Minimum Zone (OMZ) occurring in that area (Lindgren 2010). However, there are no near-shore records of this species between the mouth of the Gulf of California and Nicaragua. De Silva-Dávila et al. (2013) processed pelagic samples from the entire Gulf of California, but found *Pterygioteuthis hoylei* only in the central and southern portions of the Gulf, with a strong affinity for the area south of ca. 28°N. In our study, *Pterygioteuthis hoylei* was not collected north of ca. 26°N. Our sampling effort from off the west coast of Mexico (roughly from 16°49 to 19°30'N, in 27 stations) was negative and not a single specimen of *Pterygioteuthis* was collected there. Lindgren (2010) referred to the possibility that *Pterygioteuthis hoylei* might take advantage of the boundary effect of the OMZ, a very strong and characteristic feature of this area in the eastern Pacific Ocean (Díaz and Rosenberg 1995; Helly and Levin 2004; Serrano 2012), finding abundant food items like large zooplanktonic organisms. Distribution maps of *Pterygioteuthis hoylei* in the Gulf of California presented by Lindgren (2010) and De Silva-Dávila et al. (2013) somehow matches the area of strong upwellings, while its distribution off Central America corresponds to the area of influence of the Costa Rica Dome, a highly productive zone that is also a favorite destination for marine mammals (Rosales-Nanduca et al. 2011). The OMZ core along the SW coast of Mexico is very ample, on the average covering a depth range of >700 m (Hendrickx and Serrano 2010). In shallow water, the well oxygenated fringe in this area is very narrow (<50 m) (Hendrickx and Serrano 2010), hence small squids like *Abraliopsis* and *Pterygioteuthis* probably occur below the OMZ core. There is no evidence of these species being able to migrate throughout the OMZ which features a severely hypoxic-anoxic (<0.2 ml/l O_2_) central core between ca. 50–100 m and 1000 m (Hendrickx and Serrano 2010). In the western Atlantic *Pterygioteuthis hoylei* lives between 400 to 500 m and probably migrates to 200 m depth during the night (Lu and Roper 1979). In California it has been reported between 300 and 600 m (Roper and Young 1975).

Although almost 50 samples of benthic organisms were obtained from off the west coast of the Baja California Peninsula between June 2012 and June 2014, with the benthic sledge operating at similar depth as those reported herein (see material examined), not a single specimen of pelagic squid was found. There is no clear explanation for this. Lindgren (2010) reported the presence of *Pterygioteuthis gemmata* off the Baja California Peninsula roughly north of 28°N. *Pterygioteuthis gemmata* tends to show low densities and a more northern distribution (Lindgren 2010) than the other species. Our specimens are juveniles, but their photophores pattern and other anatomical features fit well with *Pterygioteuthis gemmata* (Lindgren, 2011). The distribution area for the other species in the genus does not include the California Current area (Lindgren 2010). *Abraliopsis affinis* has been reported from 20°N to 30°S, including off Mexico, while *Abraliopsis falco* has been collected between 35°N (southern California) and 20°S, also including Mexico in its distribution range (Jerep and Roper 2010).

As for many marine taxa with wide distribution, identification and congruence of the morphological characters need to be reinforce using molecular markers; unfortunately oegopsids do not show high representativity in molecular analysis (Allcock et al. 2014).

Additional sampling using more appropriate gear that can be hauled at speed of at least 5–6 knots (e.g., large size mid-water trawl) would probably be more adequate and certainly provide larger series of specimens of these elusive organisms.
